# Estimating sorghum leaf dhurrin content using a handheld near infrared instrument

**DOI:** 10.3389/fpls.2026.1759377

**Published:** 2026-02-11

**Authors:** Kamaranga H. S. Peiris, Scott R. Bean, Chad M. Hayes, Yves Y. Emendack, Jacobo Sanchez

**Affiliations:** 1 Center for Grain and Animal Health, USDA-ARS, Manhattan, KS, United States; 2 Cropping Systems Research Laboratory, USDA-ARS, Lubbock, TX, United States

**Keywords:** deep learning models, dhurrin content estimation, near infrared spectroscopy, neural network models, sorghum

## Abstract

**Background:**

Dhurrin is a toxic cyanogenic glycoside present in sorghum as a secondary metabolite. As such, dhurrin content of plants is important when sorghum is used as a forage crop. Moreover, leaf dhurrin content may indicate pre- and post-flowering drought resistance of sorghum germplasm. Standard method of analysis by HPLC is expensive and time consuming. Therefore, a feasibility study was conducted to measure dhurrin content in sorghum leaves by NIR spectroscopy using a handheld NIR spectrometer.

**Results:**

Partial least squares (PLS) regression, back propagation neural network (BPNN) and deep learning artificial neural network (ANNDL) models were developed to estimate dhurrin content in fresh and dry sorghum leaves. However, it was impossible to develop useful models with fresh leaf scans. NIR spectroscopy models could estimate the dhurrin content of dry sorghum leaves with R^2^ = 0.711-0.719, RMSEP = 5.89 - 5.96 µg/mg and bias of -0.26 – 0.11 µg/mg in a population of leaves with dhurrin content ranging from 0.65- 46.52 µg/mg.

**Conclusions:**

Therefore, NIR spectroscopy may be used as a rapid and cost-effective technique for pre-screening sorghum germplasm for leaf dhurrin content in plant breeding programs.

## Background

Cyanogenic glycosides are a widespread group of natural plant products present in many plant species (Vetter, 2000). Dhurrin [(S)-4-hydroxymandelonitrile-D-glucopyranoside] is the major cyanogenic glycoside found in different species of sorghum used for grain and forage production. Dhurrin is involved in chemical defenses against diseases, insect pests and herbivores, regulation of some plant metabolic processes, and has osmoprotectant properties (Burke et al., 2015; Krothapalli et al., 2013; Ouma et al., 2023). Dhurrin is accumulated in all parts of a sorghum plant except in mature seed (Nielsen et al., 2016). The level of dhurrin accumulation depends on genotype, age of plants, growing temperature, drought conditions, and availability of nitrogen (Busk and Møller, 2002; Gorz et al., 1986; Oberoi and Kaur, 2020; Ouma et al., 2023). Dhurrin may constitute 6% or even more of the dry weight of young sorghum seedlings (Halkier and Møller, 1989; Kojima et al., 1979; Saunders et al., 1977). Emendack et al. (2025) showed that 13-day-old seedlings of high-dhurrin level lines grown under N-deficient condition, had higher fresh biomass than their low-dhurrin level counterpart.

Stay-green trait (Thomas and Ougham, 2014) is an important agronomic trait associated with post flowering drought resistance and grain yields. Dhurrin production may contribute to the expression of stay-green traits, however, the effect is small and mostly influenced by genotype (Emendack et al., 2017; Gruss et al., 2023). Hayes et al. (2023) noted that dhurrin contents of stay-green genotypes are 2–3 times higher than the non-stay-green lines of sorghum. Hence, dhurrin content may be used as a trait for evaluation of pre- and post-flowering drought tolerance of sorghum breeding lines (Burke et al., 2013; Emendack et al., 2018).

Sorghum is also cultivated as an important forage crop in dryland regions and used for cattle grazing. Production of the cyanogenic glucoside dhurrin is a serious limitation to sorghum’s usefulness as a forage crop (Bhat, 2019). Under certain conditions (drought, after freezing temperatures, high N-fertilization, and age of leaf) dhurrin levels can be high in sorghum plant tissues and may become lethally toxic to animals when consumed as forage (Giantin et al., 2023). Gleadow stated that it would be important to develop new, low cyanogenic varieties to ensure safe grazing and to also develop simple methods to assess the cyanide levels of plants in the field (Gleadow et al., 2016).

Cyanogenic glycosides can be analyzed by direct or indirect methods. Direct methods include extraction and separation using liquid chromatography followed by mass spectroscopy or post column cleavage. Indirect methods employ the degradation of the cyanogenic glycoside and then quantification of cyanide or glucose (Gleadow et al., 2011; Vetter, 2000). However, these methods are laborious, time consuming, and expensive. This may deter the screening of large number of sorghum breeding lines at early stages of breeding. Under such circumstances, near infrared (NIR) spectroscopy may be a potential candidate method of analysis. Fox et al. (2012) used NIR spectroscopy for ground sorghum forage samples using a benchtop Foss NIRSystem 6500 instrument and reported a PLS model with R^2^ of 0.838 and SEP of 0.047% using the 400-2500m=nm spectral range. However, for individual plant analysis with fresh or dry leaves handheld instruments that can scan single leaf samples are preferable. This will allow scanning many samples in the field or lab with minimal sample preparation. Recent developments in NIR instrumentation have made it possible to miniaturize and lower the cost of instruments. Low-cost hand-held NIR instruments work well compared to the performance of benchtop laboratory grade instruments (Peiris et al., 2023). NIR spectroscopy with hand-held instruments may provide a solution as a rapid and cost-effective screening method and it is therefore important that models be developed for the implementation of this technology.

The dhurrin molecule has C-H, C-C, C≡N, O-H, C-O-C bonds and an aromatic ring. All these bonds, except the nitrile (C≡N) bond, are infrared active (Yulvianti and Zidorn, 2021). Though the nitrile bond, in general, has a very characteristic IR absorption peak around 2250 cm^-1^, attachment of an electron-attracting oxygen atom to the α-carbon atom of the C≡N group reduce the intensity of absorption (Silverstein et al., 2005). Therefore, infrared absorption of the dhurrin nitrile bond is quenched due to the presence of oxygen on the nitrile bearing carbon (Seigler, 1975). However, since the other bonds are infrared active, dhurrin content in sorghum may be quantitatively estimated by NIR spectroscopy. Since NIR spectroscopy is a rapid method of analysis, it may be used as a rapid phenotyping tool for selecting choice germplasm for generation advancement in sorghum breeding programs.

In the present study, we used three commonly used spectroscopy data modeling approaches, Partial least squares (PLS) regression, back propagation neural network (BPNN) and deep learning artificial neural network (ANNDL) for developing calibration models. PLS model assume linear relationship between the NIR absorbance and constituent level while NN models are capable of modeling non-linear relationships (Blanco et al., 2000; Ni et al., 2014). We tested the feasibility of using near-infrared spectroscopy for estimating dhurrin content of mature sorghum leaves using a handheld NIR spectrometer.

## Methods

### Tissue samples

A sample set of 149 mature sorghum leaves (first leaf below the flag leaf) consisting of three replicates from 50 distinct sorghum varieties (**Table 1**), with varying levels of leaf dhurrin content, grown under fully irrigated sub-surface drip irrigation system, with standard fertilization and management, were harvested at anthesis from the research fields of the USDA-ARS, Cropping Systems Research Laboratory in Lubbock, Texas. Twenty leaf discs of ¼ inch in diameter were punched (using tissue J Punch, MIDCO, St. Louis, MO) along each leaf on either side of the midrib into a 2.0 ml Eppendorf safe-lock tube, placed on ice and stored in freezer at 4˚C for dhurrin quantification by HPLC analysis. Leaves were carefully and individually packed in sorghum pollination bags and shipped on ice packs from the collection site to the Center for Grain and Animal Health Laboratory, Manhattan, Kansas, for NIR scanning of leaves, which commenced within 1 day of receiving the samples. One leaf sample was damaged, thus 149 of expected 150 samples were used in studies.

**Table 1:** Historical leaf dhurrin content characterization and HPLC measured dry leaf dhurrin content of the 50 genotypes from the sorghum association panel used for model calibration to estimate dhurrin content using handheld NIR.

Genotype	^Ψ^Leaf dhurrin status	^β^Lab values µg/mg	Genotype	^Ψ^Leaf dhurrin status	^β^Lab values µg/mg
SC6	High	27.1 ± 7.15	SC309	High	16.2 ± 4.21
SC13	Low	10.1 ± 2.46	SC414	Low	8.8 ± 1.24
SC15	High	44.8 ± 2.41	SC599	High	25.7 ± 3.82
SC17	High	32.2 ± 7.21	SC701	Low	8.8 ± 1.26
SC21	Low	9.8 ± 2.77	SC702	High	17.3 ± 2.35
SC22	Low	11.9 ± 2.27	SC704	Medium	8.4 ± 0.78
SC23	Medium	24.0 ± 6.55	SC1154	High	20.3 ± 1.68
SC25	Low	6.9 ± 0.81	BOK11	High	30.7 ± 2.01
SC33	High	21.5 ± 3.83	BTx2752	High	25.5 ± 1.21
SC35	High	24.5 ± 3.29	BTx399	Low	12.0 ± 1.13
SC38	High	15.8 ± 2.47	BTx642	High	24.3 ± 2.31
SC42	High	33.6 ± 4.02	BTxARG-1	Low	6.8 ± 0.71
SC49	High	34.6 ± 5.09	CE151-262-A1	High	27.3 ± 2.34
SC51	Low	19.0 ± 1.28	HEGARI	High	18.2 ± 1.62
SC52	Low	6.1 ± 1.01	KS19	Low	3.1 ± 0.13
SC53	Low	5.1 ± 0.78	Lian Tang Ai	Low	6.7 ± 0.91
SC55	High	12.3 ± 0.46	M35-1	Low	1.7 ± 0.13
SC56	High	28.9 ± 1.27	Macia	High	20.2 ± 1.78
SC103	High	17.9 ± 1.34	P898012	Low	8.9 ± 1.15
SC108	Low	6.6 ± 1.02	R9188	High	23.5 ± 2.05
SC110	Medium	8.3 ± 1.27	RIO	Medium	16.0 ± 0.78
SC170	High	23.7 ± 5.41	RTx2783	Medium	14.1 ± 0.70
SC301	High	25.6 ± 5.24	RTx430	Medium	21.7 ± 3.21
SC303	High	17.9 ± 1.17	Tx2909	Low	1.7 ± 0.17
SC305	Medium	15.3 ± 2.21	Tx2910	Low	1.3 ± 0.14

^Ψ^Historical leaf dhurrin content characterization.

^β^Averaged dry leaf dhurrin content ± standard error, measured with HPLC.

### NIR analysis

For collecting the NIR spectra, both fresh leaves and the same leaves after drying, were scanned with the instrument equipped with a measurement collar allowing the device to touch the sample so that there was a fixed distance between the sample and collection optics. Therefore, the samples were scanned in reflectance mode. Leaf spectra were collected after folding the leaf in half two and three times (4 or 8 leaf blades) because the spectra from one or two leaf blades were flat without much spectral character, probably due to short pathlength. Six scans were taken from each fold using a battery powered handheld ‘Micro NIR’ instrument [VIAVI MicroNIR OnSite-W (VIAVI Solutions Inc., Santa Rosa, CA)]. This instrument weighed around 250 g and recorded spectra from 908–1676 nm at 6.2 nm intervals and was connected to a computer via Bluetooth for recording the collected spectra. After the fresh leaves were scanned, unfolded leaves were left to dry at room temperature inside the paper bags on the benchtop for 10 days. The dried leaves were then scanned in the same way as the fresh leaves.

A sorghum leaf moisture calibration (R^2^ = 0.97, RMSECV = 0.77%, slope = 0.95) was used to estimate the moisture content of leaves at the time of scanning. Using dry weight basis dhurrin content quantified by HPLC and NIR predicted moisture content of leaves, dhurrin content of leaves at the time of scanning was estimated for use with respective spectra for developing and testing calibrations.

### HPLC analysis

Dhurrin was extracted from dried leaf punches in 1 ml of 80% Ethanol. Briefly, samples were vigorously vortexed upon the addition of the Ethanol and incubated at 60 °C for 60 minutes on a Labnet AccuBlock™ digital dry bath heating block (Labnet International, Cary, NC). The extract was centrifuged at 10,000 rpm for 10 minutes and 200μL of supernatant was transferred into a clean 2.0mL microcentrifuge tube. The supernatant was then dried by vacuum centrifugation in a CentriVap system (Labconco Corporation, St. Louis, MO) set to a low drying rate. Dry extracts were re-suspended in 400µL HPLC -grade water (Fisher Scientific™, Hampton, NH) by placing a zinc-plated metal bead (Daisy^®^ BBs, Daisy Outdoor Products, Rogers, AK) in the 2.0mL microcentrifuge tube containing the dried extract and mix-shaking for 1 minute at 30 cycles s^-1^ on a TissueLyzer^®^ mill (Qiagen, Hilden, Germany). Samples were then centrifuged at 11,000 rpm for 15 minutes to pelletize any particulates and 75µl of supernatant carefully pipetted into properly labeled vials for HPLC analysis. Sugar separation analysis was performed using a 100 x 7.8-mm Rezex™ RCM Monosaccharide Ca^2+^ (8%) ion-exchange column (Phenomenex^®^, Torrance, CA) heated to 85 °C and HPLC-grade water (Fisher Scientific™, Hampton, NH) as the mobile phase with a flow rate of 0.525ml min^-1^. A Prominence Series HPLC system fitted with an evaporative light scattering detector (Shimadzu North America, Carlsbad, CA) was used for data collection and analysis. The presence of dhurrin was determined based on the retention time in comparison to the commercial standard (Sigma-Aldrich, St. Louis, MO). Dhurrin amount (microgram - µg) was calculated by use of a log-log linear calibration curve of commercial dhurrin standard amounts (microgram - µg) injected into the HPLC system for analysis. The calibration curve was prepared from the following injected amounts of dhurrin: 7µg, 5µg, 3µg, 1µg, 0.5µg and their respective peak height. A log-log linear fit was fitted through the data with an R^2^ of 0.99933, a slope of 1.48769, and average deviation of the standards was 2.26%. The final dhurrin concentration was then determined by dividing the calculated amount (µg) in the injected sample by the equivalent dry weight (milligram – mg) of the injected sample.

### Spectral data analysis

For this feasibility study, each leaf was considered as an independent sample. Dhurrin concentrations of 100 leaves were used for calibration development and 49 leaves for validation with two replicated scans per leaf. Replicate 1 consisted of the average of 6 scans taken from 4 leaf blades while replicate 2 was collected from 8 leaf blades. Each leaf was folded 2 and 3 times, to get 4 and 8 leaf blades for scanning, respectively. Calibration and validation samples were selected manually after sorting the samples based on dhurrin levels and then selecting first two samples for calibration set and third sample for validation. In doing so, we had the calibration set with the highest dhurrin range followed by the validation set having a slightly narrower dhurrin range while both calibration and validation sets had nearly equal dhurrin distribution.

Solo (Eigenvector Research, Manson, WA) software was used to build NIR models. Calibration models were developed using the average spectra of leaves and the respective dhurrin concentration on fresh weight basis.

A total of 35 PLS models were built for comparison with seven spectral data preprocessing methods [Autoscale, Mean center, Multiplicative Scatter Correction (MSC)/Mean center, Extended Multiplicative Scatter Correction (EMSC)/Mean center, Standard Normal Variate (SNV)/Mean center, Savitzky–Golay (SG) second order polynomial first derivative with 15 data points and SG second order polynomial second derivative with 15 data points] in combination with five number of latent variables (4, 5, 6, 7 or 8) using the Model Optimizer tool of the Solo software. Likewise, 63 BPNN models (Seven preprocessing methods as stated above in combination with three (2, 3 or 4) nodes for the first hidden layer and three X-block (Spectral data) compression (None or X-block compression with 12 PCA or PLS components) were built and compared. Scikit Learn framework with 5 nodes for the first layer with ‘lbfgs’ solver and ‘relu’ activation function was used to develop 21 ANNDL models with the combination of the above seven preprocessing methods with three X-block compression (None, 12 PCA or PLS X-block compression components). Optimum models were selected based on RMSEP, R^2^ of prediction and RMSECV/RMSE ratio.

## Results

**Figure 1:**
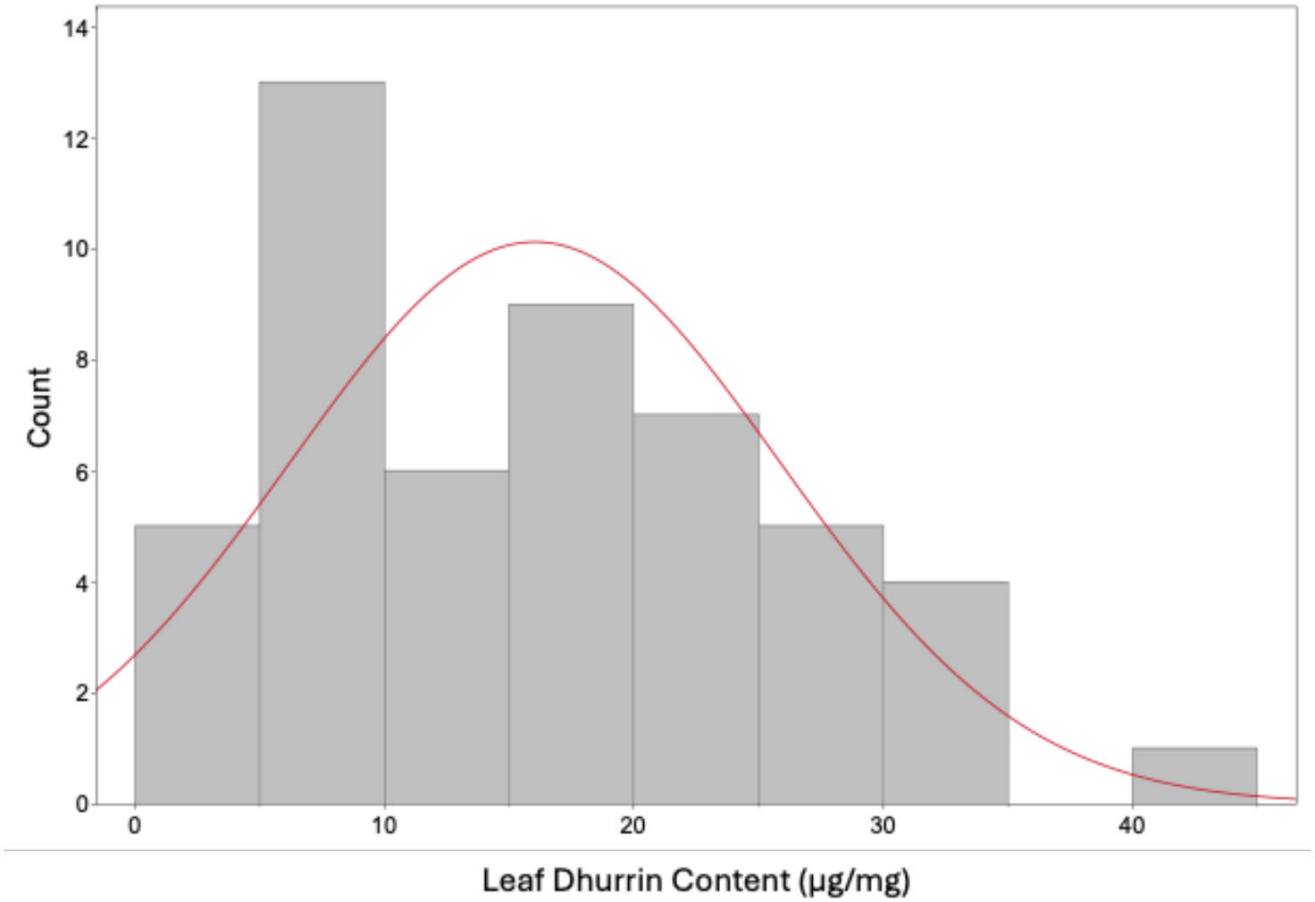
Distribution of samples showing the variation in dry leaf dhurrin content.

HPLC analysis of dry leaf samples confirmed the varying levels of leaf dhurrin content of the 50 genotypes from the sorghum association panel used for the model calibration (**Figure 1**).

Results are presented for the selected models built with dry leaf scans. The calibration models built with NIR scans from fresh leaves did not yield useful models, likely due to masking of the dhurrin absorption bands by strong moisture bands (Büning-Pfaue, 2003; Moll et al., 2022) due to the high moisture content of fresh sorghum leaves.

Dhurrin concentrations of the calibration samples ranged from 0.50 - 55.30 µg/mg on fresh weight basis while the validation set dhurrin concentrations ranged from 0.65 - 46.54 µg/mg (**Table 2**).

**Table 2:** Descriptive statistics of dhurrin concentration (µg/mg) in leaf samples.

Sample set	N *	Min	Max	Avg	STD
Calibration set	100	0.50	55.30	14.80	11.77
Validation set	49	0.65	46.54	14.48	11.04

*N, Number of samples; Min, minimum; Max, maximum; Avg, average; STD, standard deviation.

The moisture content of dry leaves was between 2.51-13.04% with a mean of 8.41% and standard deviation of 1.73%.

**Figure 2:**
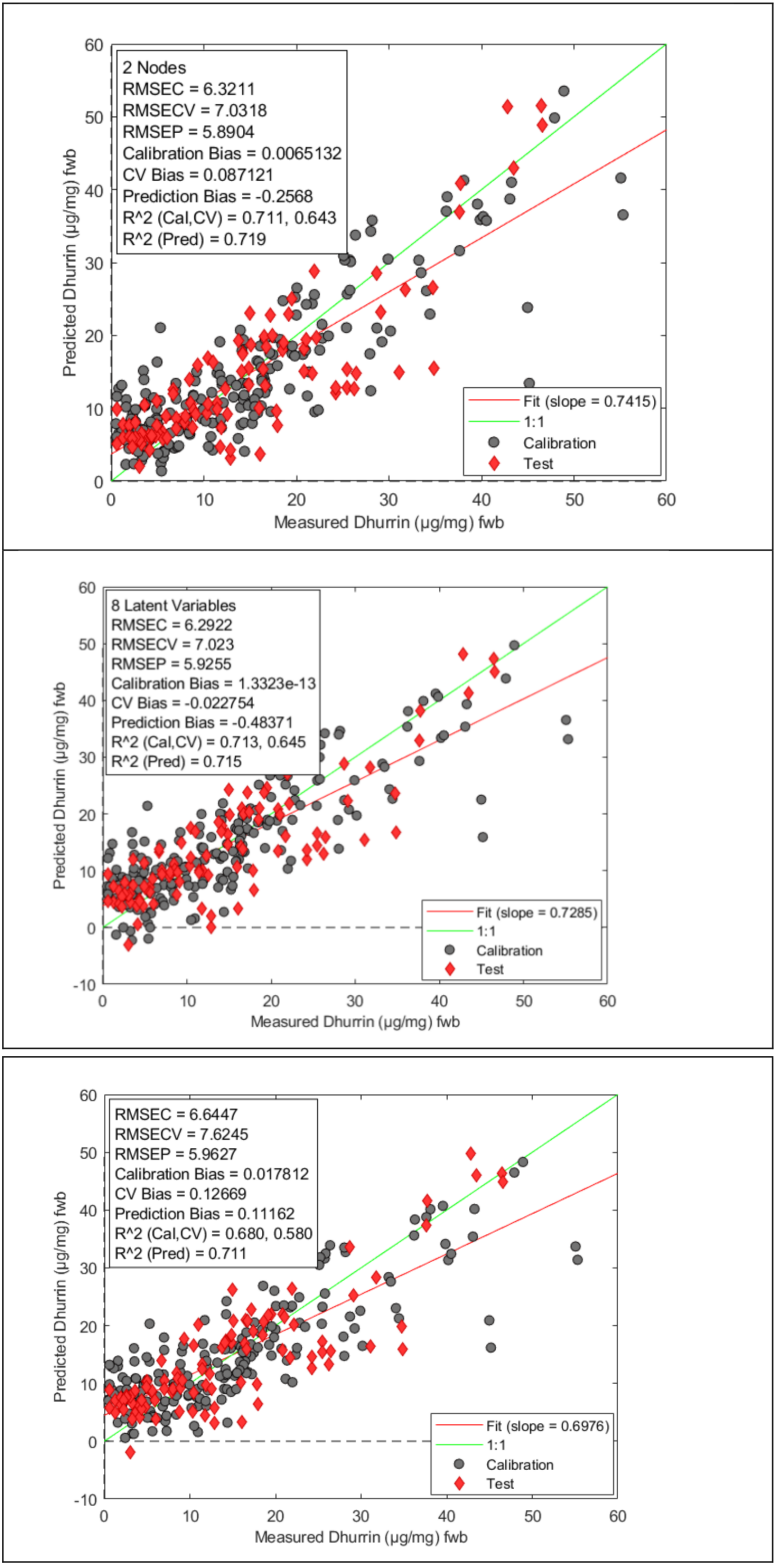
Measured dhurrin content vs predicted dhurrin content for the BPANN (Top), PLS (middle) and ANNDL (bottom) models.

Calibration and validation results for the BPNN, PLS and ANNDL models are shown in **Figure 2**. All selected models had EMSC with Mean centering as the preprocessing method showing that EMSC/Mean center is the choice preprocessing method among the seven methods tested for this dataset. BPNN model having one hidden layer with two nodes using 12 compressed PCA components of the spectral data matrix predicted the dhurrin concentration of the validation samples with R^2^ = 0.719, RMSEP = 5.89 µg/mg and bias of -0.26 µg/mg. The PLS model with 8 Latent Variables predicted the validation set with R^2^ = 0.715, RMSEP = 5.93 µg/mg with a bias of -0.48 µg/mg. Respective validation statistics for the ANNDL model that used EMSC/Mean center preprocessed spectral data without PCA or PLS compression was R^2^ = 0.711, RMSEP = 5.96 µg/mg with a bias of 0.11 µg/mg. These results show that all three models predicted the dhurrin levels in a nearly similar manner.

## Discussion

The performances of these models show that NIR spectroscopy may be used for estimating dhurrin concentrations of dry sorghum leaves. All three models predicted the dhurrin levels in dry sorghum leaves in nearly similar manner with very slight changes in R^2^ and RMSEP. Considering that dhurrin levels as low as 0.0 µg/mg and as high as 60 µg/mg or more found have been detected in sorghum leaves (Halkier and Møller, 1989; Emendack et al., 2018, 2025; Hayes et al., 2023) from diverse panels of sorghum genotypes, the 0.5 to 55.3 µg/mg reported by the models in this study can be used satisfactorily to identify samples as low, medium and high dhurrin samples. This will allow users to pre-screen and select the interested plants with high or low dhurrin levels for further analysis resulting in savings of cost and time of analysis. Standard methods to determine dhurrin content are expensive and laborious. For example, analysis of dhurrin content in one leaf sample by HPLC method will take about 2 hrs. and around $75 - $250/sample depending on whether lab is academic or commercial. This may inhibit the evaluation of large plant populations such as early breeding populations. With this NIR method dhurrin levels could be estimated in air dried leaf samples in triplicate in less than two minutes. Therefore, this method could be employed as an effective rapid screening method for large number of samples and thus help streamlining evaluation of individual plants in large breeding populations.

For future use, these NIR spectroscopy models would need to be further improved by adding additional samples grown from different locations and seasons as well as adding leaf samples from dhurrin free mutants to young leaves of high dhurrin varieties with a view to extending the expected range of dhurrin content in sorghum leaves at different stages of growth. NIR spectroscopy has the potential to be useful as a cost-effective high throughput pre-screening method for evaluation of dhurrin content in sorghum germplasm in breeding experiments using dried leaves. Gorz et al. (1986) and Gleadow et al. (2012) showed that dhurrin is stable in dried sorghum plant materials. Therefore, drying leaves will not affect the results whether fresh or dried leaves are used for analysis.

The ability to estimate dhurrin content of fresh *in situ* plant samples in the field has a definite advantage in that it is possible to get dhurrin content nondestructively as the plants grow allowing one to study changes in dhurrin with age of plants. This may also allow rapid assessment of dhurrin level in the field before the fields are allowed for grazing. NIR region in the 950 to 1650 spectral range has very broad and strong water bands resulting from a composite of overtone and combination bands of water (Büning-Pfaue, 2003). Strong water bands can obscure the NIR absorption bands of constituents found at lower concentrations (Moll et al., 2022), especially when the moisture content of the sample is high. Moreover, infrared band of the nitrile bond of dhurrin is quenched (Saunders et al., 1977). Hence, NIR spectroscopy has a disadvantage for estimating dhurrin content of fresh sorghum tissues. Raman spectroscopy may be a more suitable method for quantifying dhurrin in fresh sorghum plants in the field. Heraud et al. (2018) showed that the nitrile bond of dhurrin molecule is Raman active with a characteristic sharp peak around 2245cm^-1^ (Heraud et al., 2018). Since water does not interfere with Raman spectra (Rostron et al., 2016), Raman spectroscopy may be a more suitable method for assessing dhurrin content of fresh leaves or stems of sorghum plants in the field. Since handheld Raman instruments are available now (Jehlicka and Culka, 2022; Pestle et al., 2015) it may be worthwhile to test as an alternative method for rapid dhurrin analysis of plants in the field.

The performance of NIR methods is evaluated using statistics such as RMSEP, R^2^ and Bias. Suitability of a NIR method for a particular purpose is primarily determined by RMSEP and the expected range of constituents in the sample populations. Reported models in this study with R^2^ = 0.711-0.719, RMSEP = 5.89 - 5.96 µg/mg and bias of -0.26 – 0.11 µg/mg could be able to measure dhurrin levels of a sample population with known variability range of 0- 60 µg/mg of dhurrin sufficiently enough to identify plants at least with high and low dhurrin levels. Therefore, application of this method can help reduce the costs of analysis. Since this is a rapid method of analysis, samples can be analyzed with 2–3 replicates to get a better estimate of the constituent. Considering the RMSEP of around ~ 6 µg/mg, this may not be used for applications involving thorough quality control of samples if a higher accuracy is required.

## Conclusions

The feasibility of using a handheld NIR spectroscopy instrument was tested to estimate the dhurrin content of sorghum leaves. Development of useful models with fresh leaf NIR scans were not successful. Dhurrin concentrations of dry sorghum leaves were estimated with a RMSEP of 5.89 µg/mg dhurrin using a BPNN model. PLS and ANNDL models also predicted Dhurrin levels well with RMSEP of 5.93 and 5.96 µg/mg, respectively. These results show that NIR spectroscopy may be used for estimating dhurrin content in dry sorghum leaves. This will allow researchers to identify germplasm with high or low dhurrin content in a timely, less expensive, and non-destructive manner.

## Data Availability

The raw data supporting the conclusions of this article will be made available by the authors, without undue reservation.
